# Body Roundness Index and Incident Colorectal Cancer Risk: A Nationwide Population-Based Cohort Study

**DOI:** 10.3390/cancers18152466

**Published:** 2026-07-31

**Authors:** Hyun Ho Kim, Changhyeok An, Kyu-na Lee, Kyung-do Han, Kang Woong Jun

**Affiliations:** 1 Division of Colorectal Surgery, Department of Surgery, Bucheon St. Mary’s Hospital, College of Medicine, The Catholic University of Korea, 327 Sosa-ro, Bucheon-si 14647, Republic of Korea; hhkim83@hanmail.net (H.H.K.); achcolo@catholic.ac.kr (C.A.); 2 Department of Preventive Medicine and Public Health, College of Medicine, The Catholic University of Korea, Seoul 06591, Republic of Korea; 3 Department of Statistics and Actuarial Science, Soongsil University, 369 Sangdo-ro, Dongjak-gu, Seoul 06978, Republic of Korea; hankd917@naver.com; 4 Division of Vascular and Transplant Surgery, Department of Surgery, Bucheon St. Mary’s Hospital, College of Medicine, The Catholic University of Korea, 327 Sosa-ro, Bucheon-si 14647, Republic of Korea

**Keywords:** body roundness index, colorectal cancer, visceral fat, abdominal obesity, cohort study, Korea

## Abstract

**Background/Objectives:** Colorectal cancer (CRC) is among the most common cancers in Korea, and visceral adiposity is an established modifiable risk factor. The body roundness index (BRI), derived from waist circumference and height, estimates visceral adipose tissue more accurately than the body mass index (BMI) alone. We investigated the longitudinal association between the BRI and incident CRC in a large Korean national cohort. **Methods:** A nationwide cohort of 4,492,697 adults aged ≥20 years who participated in the 2012 Korean National Health Insurance Service health check-up was followed through December 2023 (mean 10.32 years). BRI values were categorized into quartiles (Q1–Q4). After applying a 1-year lag period, Cox proportional hazards regression estimated hazard ratios (HRs) and 95% confidence intervals (CIs), adjusting for age, sex, smoking, alcohol consumption, exercise, income, diabetes, hypertension, hypercholesterolemia, and chronic kidney disease. Subgroup and site-specific analyses were also performed. **Results:** During 46,382,686 person-years of follow-up, 57,644 incident CRCs were diagnosed. A higher BRI was independently and dose-dependently associated with CRC risk (Q4 vs. Q1: aHR 1.104, 95% CI 1.075–1.135; *p* for trend < 0.001). The association was significantly stronger in men (Q4 aHR 1.352, 95% CI 1.302–1.404) than in women (Q4 aHR 0.891, 95% CI 0.858–0.925; *p* for interaction < 0.001) and most pronounced in middle-aged adults (40–64 years). Site-specific analyses revealed a proximal-to-distal gradient: proximal colon (Q4 aHR 1.363), distal colon (Q4 aHR 1.244), and rectum (Q4 aHR 1.070). **Conclusions:** A higher BRI is independently and dose-dependently associated with increased CRC incidence in Korean adults, particularly in men and those with general obesity. The BRI may serve as a practical complement to the BMI for CRC risk stratification in populations with a high prevalence of metabolic obesity.

## Simple Summary

Colorectal cancer is one of the most common cancers worldwide, and abdominal obesity is a known risk factor. The body roundness index (BRI) is a simple measurement calculated from a person’s waist circumference and height that estimates how much fat is stored around the abdomen. Unlike the commonly used body mass index (BMI), the BRI better reflects the amount of harmful fat stored deep inside the belly. In this study, we analyzed data from more than 4.4 million Korean adults who underwent a national health check-up in 2012 and were followed for approximately 10 years. We found that people with a higher BRI had a significantly greater risk of developing colorectal cancer, particularly men and those who were overweight or obese. Interestingly, this relationship was reversed in women, which may reflect fundamental differences in how men and women store body fat. These findings suggest that the BRI, which can be easily calculated during routine health check-ups, may help identify individuals at higher risk of colorectal cancer.

## 1. Introduction

Colorectal cancer (CRC) is the third most diagnosed cancer and the second leading cause of cancer mortality worldwide [1]. In Korea, CRC is the second most prevalent cancer, and its incidence has increased sharply over the past three decades—a trend closely linked to the adoption of Westernized dietary habits and sedentary lifestyles alongside an epidemic of metabolic obesity [2,3].

Obesity is a well-established modifiable risk factor for CRC; excess adiposity promotes carcinogenesis through insulin resistance, chronic low-grade inflammation, dysregulation of adipokines (elevated leptin and reduced adiponectin levels), and activation of the insulin-like growth factor (IGF) signaling axis—all of which stimulate colorectal epithelial cell proliferation and limit apoptosis [4,5]. However, conventional anthropometric indices for identifying obesity have important limitations: the body mass index (BMI) does not capture the distribution of fat [6], and waist circumference (WC) cannot be used to distinguish subcutaneous fat from visceral fat [7].

The body roundness index (BRI), introduced by Thomas et al. in 2013, is a geometrical index derived from the WC and height that estimates body fat percentage and visceral adipose tissue (VAT) more accurately than the BMI or WC alone does [8]. A greater BRI has been associated with cardiometabolic conditions [9,10,11], and a cross-sectional National Health and Nutrition Examination Survey (NHANES) study revealed a significant positive association between the BRI and CRC [12]. However, that study was limited by its cross-sectional design and small number of individuals with CRC (n = 282). No prospective cohort study has investigated the association between the BRI and CRC in an Asian population.

We therefore conducted a nationwide prospective cohort study using Korean National Health Insurance Service (NHIS) data to investigate whether the BRI is longitudinally associated with the incidence of CRC and, if so, to characterize this association across clinically important subgroups and cancer sites.

Although a prior Korean NHIS cohort study reported an association between WC-defined abdominal obesity and CRC in this same database, dichotomous WC thresholds do not incorporate height and therefore cannot distinguish individuals with equivalent waist measurements but different body proportions [7]. The BRI, by combining WC and height in a geometrical model, provides a continuous height-adjusted estimate of visceral adiposity that is not captured by WC thresholds alone.

## 2. Materials and Methods

### 2.1. Study Population

This nationwide retrospective cohort study involved data from the Korean NHIS, which provides mandatory universal health coverage to approximately 97% of the Korean population and is responsible for conducting standardized biennial health check-up examinations for all enrolled individuals. The NHIS health check-up program includes comprehensive anthropometric measurements, laboratory assessments, and standardized, self-administered questionnaires on health-related behaviors. Physical examinations include measurements of height, weight, BMI, WC, and blood pressure obtained by trained examiners who undergo regular qualification reviews by the NHIS. Disease diagnoses are coded according to the Korean Classification of Diseases, 6th edition, itself based on the International Classification of Diseases, 10th Revision (ICD-10). Detailed information on the NHIS database infrastructure and data collection procedures has been described previously [13].

**Figure 1:**
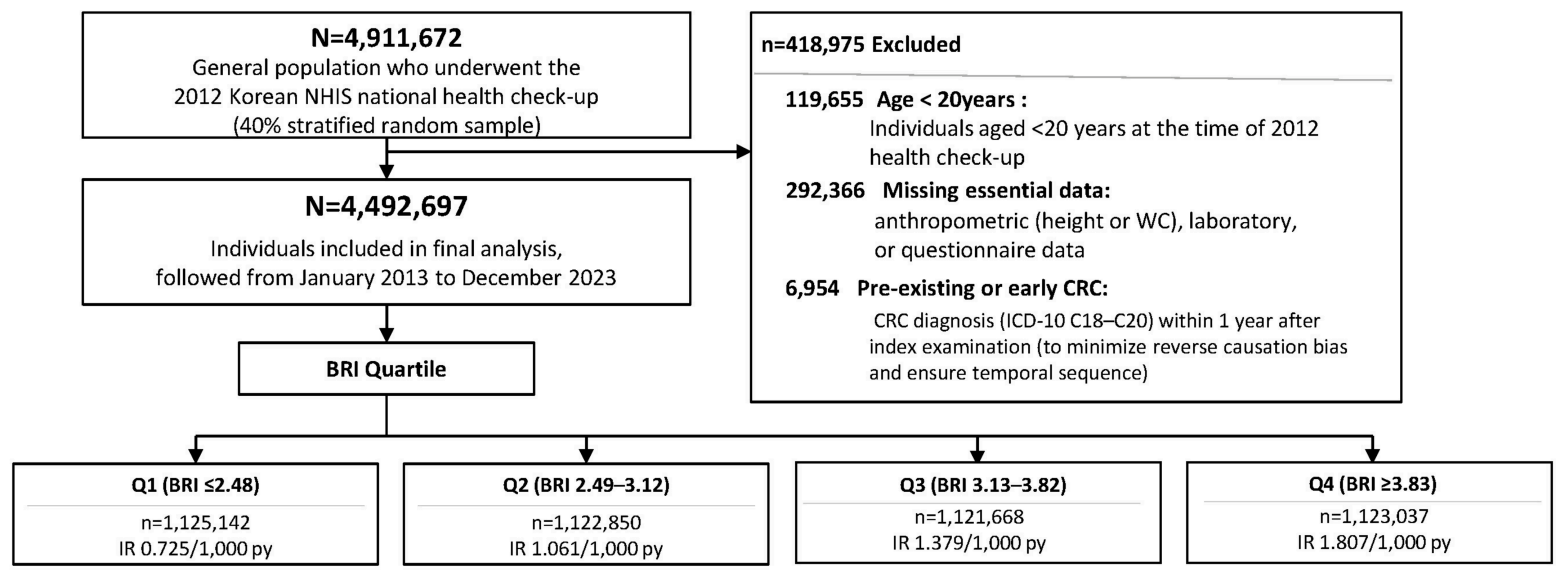
Study flow diagram. Of the 4,911,672 individuals who participated in the 2012 Korean NHIS national health check-up (a 40% stratified random sample), the following were sequentially excluded: 119,655 aged <20 years; 292,366 with missing anthropometric, laboratory, or questionnaire data; and 6954 diagnosed with CRC within the 1-year lag period applied to minimize reverse causation. The final analytic cohort comprised 4,492,697 adults, who were followed from January 2013 to December 2023 and categorized into BRI quartiles (Q1: ≤2.48; Q2: 2.49–3.12; Q3: 3.13–3.82; Q4: ≥3.83) based on the overall cohort distribution. Crude incidence rates for each quartile are shown. Log-rank *p* < 0.001 across all quartile comparisons; all six pairwise between-quartile comparisons yielded Bonferroni-adjusted *p* < 0.001. Abbreviations: BRI, body roundness index; CRC, colorectal cancer; IR, incidence rate; NHIS, National Health Insurance Service; PY, person-years.

From an initial population of 4,911,672 individuals aged ≥20 years who participated in the 2012 NHIS national health check-up (a 40% stratified random sample), we systematically applied exclusion criteria to ensure the quality of the data and minimize confounding (Figure 1). Specifically, individuals with missing anthropometric measurements (height or WC) or key laboratory or questionnaire data were excluded (n = 292,366). To ensure a focus on incident CRC vents and minimize the possibility of reverse causation, we further excluded individuals with a preexisting CRC diagnosis (ICD-10 codes C18–C20) in the NHIS cancer registry at or before the baseline examination. Additionally, participants who developed CRC within the first year after the index examination date were excluded by application of a 1-year lag period (n = 6954) to ensure an appropriate temporal sequence between exposure assessment and outcome occurrence.

After application of these exclusion criteria, the final analytic cohort included 4,492,697 participants. Follow-up commenced from 1 January 2013 (after the 1-year lag period), and continued until either (1) an incident CRC diagnosis, (2) death, or (3) the study end date of 31 December 2023, whichever came first. The mean follow-up duration was 10.32 ± 0.71 years (median 10.36 years, interquartile range 10.13–10.61 years). The study protocol was approved by the Institutional Review Board of the Catholic University of Korea, Bucheon St. Mary’s Hospital, Seoul, Korea (HC26ZISI0094). The requirement to obtain informed consent was waived because the study utilized deidentified administrative data in accordance with the Korean Personal Information Protection Act provisions for research using preexisting administrative databases. All procedures were conducted in accordance with the Declaration of Helsinki.

### 2.2. Exposure: Body Roundness Index

Height and WC were measured at the baseline health examination using standardized procedures by trained technicians. Height was measured to the nearest 0.1 cm with a stadiometer with the participant standing straight without shoes. WC was measured at the midpoint between the lower rib and the iliac crest to the nearest 0.1 cm. The BRI was subsequently calculated from these measurements using the formula originally proposed by Thomas et al. [8]:

BRI = 364.2 − 365.5 × √(1 − [WC(m)/2π]^2^/[0.5 × height(m)]^2^)

The BRI provides a two-dimensional geometric estimate of body shape and has been validated as a superior predictor of body fat percentage and VAT volume over the BMI or WC alone. Higher BRI values indicate a more circular body shape and thus greater abdominal adiposity. To enable categorical analyses and dose-response assessments, the BRI values were divided into overall quartiles on the basis of their distribution in the analytic cohort (Q1: ≤2.48, Q2: 2.49–3.12, Q3: 3.13–3.82, Q4: ≥3.83), with Q1 designated as the reference group. In a sensitivity analysis, quartiles were also computed separately by sex (Supplementary Table S9a,b). Both categorical (quartile-based) and continuous (per 1-unit increment) parameterizations were examined to assess dose-response relationships and facilitate comparisons with prior studies.

The BMI was additionally calculated as weight (kg) divided by height squared (m^2^) and categorized according to the 2018 Korean Society for the Study of Obesity guidelines for Asian populations: underweight (<18.5 kg/m^2^), normal weight (18.5–23.0 kg/m^2^), overweight (23.0–25.0 kg/m^2^), preobesity (25.0–30.0 kg/m^2^), and obesity (≥30.0 kg/m^2^). Anthropometric measurements were obtained at the baseline health examination and were not reassessed during the follow-up period.

### 2.3. Outcome: Colorectal Cancer

The primary outcome was newly diagnosed CRC, identified through the NHIS cancer registry as (1) a first-ever registration for CRC under the ICD-10 codes C18 (colon cancer), C19 (rectosigmoid junction cancer), or C20 (rectal cancer) in the NHIS cancer registration program or (2) a first-ever hospitalization with CRC as the primary discharge diagnosis. The NHIS cancer registration program in Korea mandates the reporting of all newly diagnosed cancers by treating physicians, with registration linked to a dedicated copayment reduction benefit for cancer patients, providing strong incentives for complete and accurate registration. This registration-based outcome assessment has been shown to have high sensitivity and specificity for major cancer diagnoses in the Korean NHIS.

Secondary outcomes included site-specific CRCs: proximal colon cancer (C18.0 [caecum], C18.1 [appendix], C18.2 [ascending colon], C18.3 [hepatic flexure], C18.4 [transverse colon]), distal colon cancer (C18.5 [splenic flexure], C18.6 [descending colon], C18.7 [sigmoid colon], C19.0 [rectosigmoid junction]), and rectal cancer (C20.0). Patients for whom the cancer was in an unspecified (C18.9) or overlapping (C18.8) sites were excluded from the site-specific analyses but included in the analyses of overall CRC. This anatomical subclassification was prespecified on the basis of prior evidence suggesting differential pathways of carcinogenesis between proximal and distal CRC.

In this cohort, 26,768 CRC cases (46.4%) were coded as C18.9 (unspecified location) or C18.8 (overlapping lesions), and were excluded from the site-specific analyses. This proportion reflects the coding characteristics of Korean NHIS claim data, in which anatomical subsite is not always specified for reimbursement, particularly for outpatient, emergency, or non-surgical inpatient claims. The site-specific analyses should therefore be interpreted as representative of cases with sufficient coding granularity for subsite classification rather than of all incident CRCs.

### 2.4. Covariates

Covariates were selected a priori on the basis of established evidence of associations with the risk of CRC and potential confounding effects on the BRI–CRC relationship. All covariates were ascertained at the baseline health examination.

Sociodemographic variables included age (continuous, years), sex (male/female), and household income level, the last of which was dichotomized as low (lowest quartile of national income or medical aid beneficiary [MAB]) versus high (income quartiles 2–4).

Lifestyle variables were assessed using the NHIS standardized self-administered questionnaire. Smoking status was categorized as never smoker, former smoker, or current smoker. Alcohol consumption was categorized as none, mild (<30 g of ethanol per day), or heavy (≥30 g/day), consistent with the categorization in previous NHIS-based studies [REF]. Participation in regular exercise was defined as engagement in vigorous physical activity for ≥20 min ≥3 days per week or moderate physical activity for ≥30 min ≥5 days per week.

Metabolic and clinical comorbidities were defined as follows. Diabetes mellitus was defined as fasting blood glucose ≥ 126 mg/dL at the baseline health check-up or at least one prescription claim for antidiabetic medications or insulin under ICD-10 codes E11–E14 in the 12 months prior to the index date. Hypertension was defined as a systolic blood pressure ≥ 140 mmHg or a diastolic blood pressure ≥ 90 mmHg at baseline or at least one prescription claim for antihypertensive medications under ICD-10 codes I10–I13 or I15 in the prior 12 months. Hypercholesterolemia was defined as a total serum cholesterol level ≥ 240 mg/dL at baseline or the presence of ICD-10 code E78 alongside a prescription claim for lipid-lowering medications in the prior 12 months. Chronic kidney disease (CKD) was identified on the basis of an estimated glomerular filtration rate (eGFR) < 60 mL/min/1.73 m^2^, which was calculated from serum creatinine using the Modification of Diet in Renal Disease (MDRD) equation. BMI was additionally included as a covariate in the sensitivity analyses to assess whether the associations between BRI and CRC were independent of overall adiposity.

### 2.5. Statistical Analysis

The baseline characteristics of the participants are presented as the mean ± standard deviation (SD) for continuous variables and as frequencies with percentages for categorical variables. Differences across BRI quartiles were assessed using one-way analysis of variance (ANOVA) for continuous variables and the chi-square test for categorical variables. All reported *p* values for between-group comparisons were <0.001.

The incidence of CRC was calculated as the total number of incident CRC events divided by the total person-years at risk within each BRI quartile group, expressed as cases per 1000 person-years. The temporal distribution of CRC events was assessed using the Kaplan–Meier method; cumulative incidence curves were stratified by BRI quartile, and between-group differences were evaluated with the log-rank test.

Associations between the BRI and incidence of CRC were estimated using Cox proportional hazards regression, and the results are expressed as hazard ratios (HRs) and 95% confidence intervals (CIs). Three hierarchical models were constructed: Model 1 was unadjusted; model 2 was adjusted for age (continuous) and sex; and model 3 (fully adjusted) was further adjusted for smoking status, alcohol consumption, participation in regular exercise, income level, diabetes mellitus status, hypertension status, hypercholesterolemia status, and CKD status. These covariates were selected to reflect the major established determinants of the risk of CRC and potential confounders of the relationship between the BRI and CRC. The proportional hazards assumption was evaluated by inspecting log-log survival plots and testing for nonzero slopes in Schoenfeld residual regressions against follow-up time for all covariates; no significant violations were identified. Models 1–3 represent a prespecified sequence of hierarchical confounder adjustment (Model 1: unadjusted; Model 2: age- and sex-adjusted; Model 3: fully adjusted), presented for descriptive transparency rather than as competing candidate models for model selection.

Prespecified subgroup analyses were conducted to examine whether the associations between BRI and CRC were affected by age group (<40, 40–64, ≥65 years), sex, household income level, smoking status, alcohol consumption, participation in regular exercise, BMI category (<25 vs. ≥25 kg/m^2^), diabetes mellitus status, hypertension status, hypercholesterolemia status, and CKD status. For each subgroup analysis, the BRI was analyzed in categorical (quartile) format, with Q1 used as a reference. Tests for statistical interaction were performed by introducing multiplicative interaction terms (BRI quartile × subgroup indicator) into the fully adjusted Cox model. *p* values for interaction less than 0.05 were considered indicative of an effect modification.

Site-specific CRC analyses (proximal colon, distal colon, and rectum) were conducted using the same Cox regression framework, in which deaths and non-index-site CRC diagnoses were censoring at time of occurrence in the primary Cox regression (cause-specific hazard approach). As a sensitivity analysis, a Fine–Gray subdistribution hazard model was additionally fitted, in which all-cause mortality and non-index-site CRC diagnoses were formally treated as competing events (Supplementary Table S8).

A two-tailed *p* value < 0.05 was considered to indicate statistical significance. *p* values were not adjusted for multiplicity given the prespecified nature of the subgroup and sensitivity analyses. The selection of the study population is illustrated in Figure 1. Briefly, after excluding participants aged <20 years (n = 119,655), those with missing anthropometric, laboratory, or questionnaire data (n = 292,366), and those diagnosed with CRC within the 1-year lag period (n = 6954), the final analytic cohort comprised 4,492,697 individuals. All the statistical analyses were performed using SAS software, version 9.4 (SAS Institute Inc., Cary, NC, USA).

Complete-case analysis was adopted as the primary approach; the excluded 292,366 individuals with missing baseline data constituted 5.9% of the initial cohort, consistent with standard analytical practice in prior large-scale NHIS-based studies. Multiple imputation was not applied because the conditional missing-at-random assumption cannot be reliably verified in administrative databases of this scale, and because missingness in NHIS check-up data most plausibly arises from non-attendance at individual measurement stations rather than disease-related mechanisms.

## 3. Results

### 3.1. Baseline Participant Characteristics

The study flow diagram is presented in Figure 1. A total of 4,492,697 participants were included in the final analytic cohort, with a mean age of 48.05 ± 13.77 years, and 54.34% were male. The distribution of the BRI quartile groups was approximately balanced (Q1: n = 1,125,142; Q2: n = 1,122,850; Q3: n = 1,121,668; Q4: n = 1,123,037). The BRI quartile ranges were as follows: Q1 ≤ 2.48, Q2 2.49–3.12, Q3 3.13–3.82, and Q4 ≥ 3.83 (Table 1).

The baseline participant characteristics varied significantly across BRI quartile groups (all *p* < 0.001). Participants in the highest BRI quartile (Q4) were substantially older (mean age 55.34 ± 13.54 years vs. 40.20 ± 11.98 years) and had markedly greater BMIs (27.05 ± 3.01 vs. 20.57 ± 1.91 kg/m^2^) and WC (90.43 ± 6.70 vs. 69.72 ± 5.20 cm) than those in BRI Q1 were. As the BRI increased, the prevalence of abdominal obesity (Q1: 0.00% vs. Q4: 69.87%), diabetes mellitus (1.83% vs. 11.66%), hypertension (4.77% vs. 24.42%), hypercholesterolemia (5.44% vs. 15.64%), and CKD (1.66% vs. 6.79%) increased monotonically. Cardiometabolic laboratory values—including systolic and diastolic blood pressure, fasting plasma glucose level, total cholesterol level, low-density lipoprotein (LDL) cholesterol level, and triglycerides—worsened progressively with increasing BRI, whereas the high-density lipoprotein (HDL) cholesterol level and eGFR decreased (all *p* < 0.001). The percentage of never smokers was elevated at both extremes of the BRI distribution (Q1: 67.21%; Q4: 62.16%), a pattern that likely reflects compositional differences in age and sex across the quartile groups rather than a monotonic trend with BRI. Regular exercise participation was similar across quartiles (range 17.14–20.44%).

### 3.2. BRI and Overall Incidence of Colorectal Cancer

**Figure 2:**
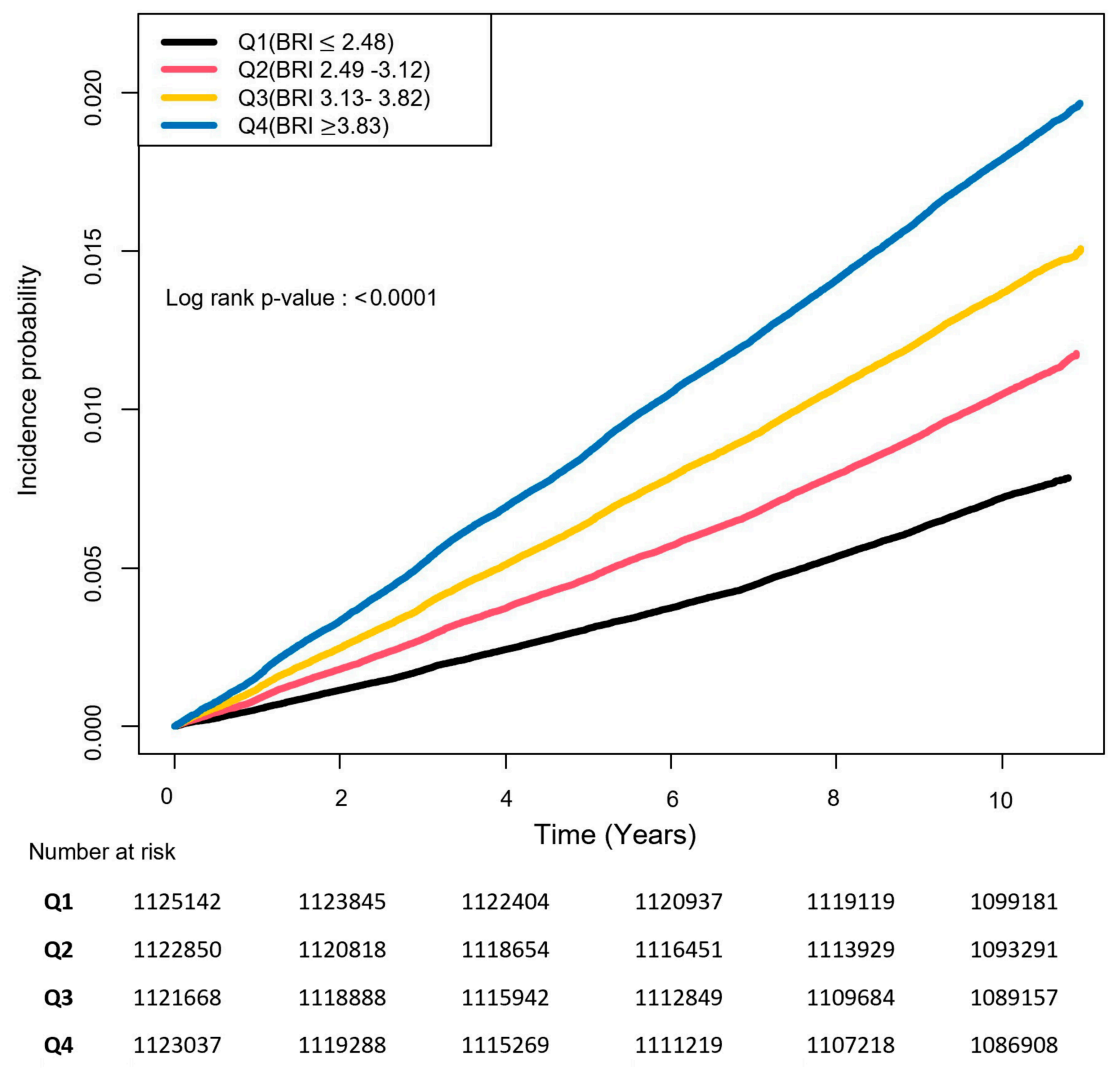
Cumulative incidence of CRC according to body roundness index (BRI) quartile among 4,492,697 Korean adults (2012–2023). Cumulative incidence curves are shown for the BRI quartile groups: Q1 (BRI ≤ 2.48, black solid line), Q2 (BRI 2.49–3.12, red dashed line), Q3 (BRI 3.13–3.82, yellow dashed-dotted line), and Q4 (BRI ≥ 3.83, blue solid line). The number of participants at risk at each annual time point is presented below the curves. Follow-up commenced after a 1-year lag period from the baseline health examination. Pairwise log-rank tests with Bonferroni adjustment for multiple comparisons yielded *p* < 0.001 for all six between-quartile pairs (Q1–Q2, Q1–Q3, Q1–Q4, Q2–Q3, Q2–Q4, Q3–Q4). Abbreviations: BRI, body roundness index; CRC, colorectal cancer; IR, incidence rate; py, person-years.

During the 46,382,686 total person-years of follow-up (mean 10.32 ± 0.71 years per participant), 57,644 incident cases of CRC were diagnosed. The overall crude incidence was 1.243 per 1000 person-years. The crude incidence increased progressively and monotonically across the BRI quartiles (all per 1000 person-years): 0.725 in Q1, 1.061 in Q2, 1.379 in Q3, and 1.807 in Q4. Kaplan–Meier analysis revealed a progressively greater cumulative incidence of CRC with increasing BRI (Figure 2); the results of the log-rank test confirmed a statistically significant difference among the quartile-specific curves (*p* < 0.001). The number of participants at risk at each annual time point is provided in Supplementary Table S1.

Cox proportional hazards regression analysis revealed a consistent, dose-dependent, positive association between the BRI quartile and the risk of CRC across both adjusted models (Table 2). In the unadjusted Model 1, the HRs for Q2, Q3, and Q4 relative to Q1 were 1.463 (95% CI 1.423–1.505), 1.902 (1.853–1.953), and 2.492 (2.430–2.556), respectively. After adjustment for age and sex (Model 2), the HRs were substantially attenuated but remained significant: Q2 1.044 (1.015–1.074), Q3 1.093 (1.064–1.123), and Q4 1.147 (1.116–1.178). In the fully adjusted model (Model 3), which additionally incorporated smoking status, alcohol consumption, participation in regular exercise, income, diabetes mellitus status, hypertension status, hypercholesterolemia status, and chronic kidney disease status, the HRs were 1.030 (95% CI 1.002–1.059) for Q2, 1.067 (1.038–1.097) for Q3, and 1.104 (1.075–1.135) for Q4 (*p* for trend < 0.001). The stepwise attenuation in the effect estimates from Model 1 to Model 3 reflects the partial confounding by metabolic comorbidities and lifestyle factors, while the persistence of the significant associations in Model 3 confirms an independent relationship between the BRI and the risk of CRC.

### 3.3. BRI and Site-Specific Colorectal Cancer Risk

Site-specific analyses revealed that the BRI was positively and significantly associated with the risk of CRC at all three evaluated anatomical sites, although with markedly different effect magnitudes (Supplementary Table S2). For proximal colon cancer (ICD-10 C18.0–C18.4), the fully adjusted HRs for Q2, Q3, and Q4 vs. Q1 were 1.161 (95% CI 1.063–1.268), 1.240 (1.139–1.349), and 1.363 (1.254–1.481), respectively (*p* for trend < 0.001). For distal colon cancer (C18.5–C18.7, C19.0), the corresponding HRs were 1.027 (0.959–1.101), 1.150 (1.077–1.229), and 1.244 (1.166–1.328), respectively (*p* for trend < 0.001). For rectal cancer (C20.0), the corresponding HRs were 1.029 (0.972–1.090), 1.056 (0.998–1.116), and 1.070 (1.011–1.132), respectively (*p* for trend < 0.001). A statistically significant proximal-to-distal gradient in the BRI–CRC association was observed, with the strongest effect magnitude observed for proximal colon cancer (Q4 HR 1.363) and the weakest for rectal cancer (Q4 HR 1.070).

### 3.4. Subgroup Analyses

The results of the prespecified subgroup analyses are presented in Table 3 (age, sex, BMI, and diabetes; selected results) and Supplementary Tables S3a,b and S4 (all 11 prespecified variables). Significant interactions were detected for age group (*p* for interaction < 0.001), sex (*p* < 0.001), BMI category (*p* < 0.001), diabetes mellitus status (*p* = 0.026), smoking status (*p* < 0.001), and alcohol consumption (*p* < 0.001), indicating that the association between the BRI and CRC was meaningfully modified by these variables.

Age. The association between the BRI and CRC clearly demonstrated an age-dependent pattern. Among participants younger than 40 years, no significant association was observed (Q4 aHR 0.970, 95% CI 0.881–1.067). In contrast, in the 40–64-year-old age group, the association was strongest (Q4 aHR 1.126, 95% CI 1.089–1.164), whereas among those aged ≥65 years, the association was attenuated but remained statistically significant (Q4 aHR 1.125, 95% CI 1.060–1.194). In the simplified Q4 vs. Q1–Q3 analysis, the HR for the 40–64 year age group was 1.111 (95% CI 1.086–1.137) compared with 1.011 (0.982–1.041) for ≥65 year age group.

Sex. The most striking interaction was observed for sex (*p* for interaction < 0.001). Among men, a higher BRI was strongly and monotonically associated with an elevated risk of CRC: Q2 aHR 1.123 (95% CI 1.080–1.168), Q3 aHR 1.231 (1.186–1.278), and Q4 aHR 1.352 (1.302–1.404). In contrast, among women, a higher BRI quartile was associated with a lower CRC risk: Q2 aHR 0.974 (0.936–1.014), Q3 aHR 0.915 (0.879–0.953), and Q4 aHR 0.891 (0.858–0.925). In the simplified Q4 vs. Q1–Q3 analysis, the HR for men was 1.175 (95% CI 1.148–1.202) versus 0.926 (0.902–0.951) for women.

BMI. Among participants with a BMI < 25 kg/m^2^ (nonobese according to Asian criteria), the Q4 BRI was not associated with an elevated risk of CRC (aHR 0.986, 95% CI 0.952–1.023), whereas among those with a BMI ≥ 25 kg/m^2^, the Q4 association was significantly positive (aHR 1.212, 95% CI 0.996–1.474). The Q4 vs. Q1–Q3 analysis similarly revealed an aHR of 0.948 (0.921–0.976) in the BMI < 25 kg/m^2^ group and of 1.097 (1.065–1.131) in the BMI ≥ 25 kg/m^2^ group.

Diabetes mellitus. A significant effect modification was observed for diabetes status (*p* = 0.026). Among nondiabetic participants, the Q4 BRI was associated with an aHR of 1.109 (95% CI 1.078–1.140). Among those with prevalent diabetes, the Q4 aHR was 1.122 (1.002–1.255), suggesting a possibly stronger absolute risk attributable to a high BRI in the presence of existing insulin resistance, although the confidence intervals were wide. In the simplified analysis, however, the Q4 vs. Q1–Q3 HR among diabetic participants was 0.991 (0.944–1.041), suggesting no additional excess risk in the highest BRI quartile beyond what was already captured in the lower quartiles.

In the quartile-based parameterization, the raw *p* value for the income interaction was 0.054 and did not survive FDR correction (FDR-adjusted *p* = 0.084), whereas exercise, hypertension, hypercholesterolemia, and CKD showed no meaningful interaction (all raw and FDR-adjusted *p* > 0.10). The diabetes interaction remained statistically significant after FDR correction (raw *p* = 0.026, FDR-adjusted *p* = 0.048). The quartile-based Q4-vs.-Q1 parameterization is designated as our primary approach because it preserves the ordinal dose–response structure of the BRI. The simplified Q4-vs.-Q1–Q3 parameterization compresses information across three lower quartiles into a single reference category and can therefore yield materially different interaction estimates (for example, diabetes: quartile-based *p* = 0.026; simplified *p* = 0.004). Results from the simplified parameterization are presented in Supplementary Table S4 as supportive analyses. Among the smoking subgroups, the BRI–CRC association was absent in never smokers (Q4 aHR 0.993, 95% CI 0.960–1.028) but significant in former smokers (Q4 aHR 1.335, 95% CI 1.246–1.431) and current smokers (Q4 aHR 1.258, 1.194–1.326). Similarly, among the alcohol consumption groups, the association was absent for nondrinkers (Q4 aHR 1.019, 0.984–1.056) but significant for mild (Q4 aHR 1.234, 1.182–1.289) and heavy drinkers (Q4 aHR 1.184, 1.080–1.298).

### 3.5. Sensitivity Analyses

The primary positive Q4-vs.-Q1 association (aHR 1.104, 95% CI 1.075–1.135) was robust across multiple sensitivity analyses (Supplementary Table S7). Extending the lag period to 2, 3, and 5 years yielded Q4 aHRs of 1.108 (1.078–1.140), 1.116 (1.083–1.150), and 1.129 (1.092–1.168), respectively, arguing against reverse causation from occult prevalent malignancy. Restricting follow-up to the first 5 years yielded a slightly attenuated but still significant Q4 aHR of 1.072 (1.023–1.123). Under a Fine–Gray subdistribution hazard model that formally treated all-cause mortality and non-index-site CRC as competing events, the Q4 subdistribution hazard ratio was 1.094 (1.065–1.124), essentially identical to the cause-specific estimate. Additional adjustment for BMI as a dichotomous obesity indicator preserved the Q4 aHR (1.051, 1.018–1.085), whereas additional adjustment for continuous BMI attenuated the Q4 aHR to 0.983 (0.947–1.020); the latter reflects statistical overadjustment given the high Pearson correlation between BRI and continuous BMI (r ≈ 0.78), as discussed in Section 4.4.

## 4. Discussion

In this nationwide prospective cohort study of 4,492,697 Korean adults followed for a mean of 10.32 years, we demonstrated that a higher BRI was independently and dose-dependently associated with an increased incidence of CRC. To our knowledge, this is the largest prospective study examining the relationship between the BRI and CRC in any population and the first to do so in an East Asian cohort, for whom the patterns of body fat distribution differ substantially from those in Western populations. Our findings extend the prior NHANES-based cross-sectional evidence [12] by providing longitudinal data of 57,644 incident events—approximately 200-fold more than the 282 cases available in the NHANES study—enabling robust estimations of subgroup-specific effects that were previously underpowered.

### 4.1. The BRI as a Marker of Visceral Adiposity and CRC Risk

The dose-response relationship between the BRI and risk of CRC that we observed is mechanistically consistent with the established role of visceral adiposity in the development of CRC. Compared with the BMI or WC alone, the BRI, which is derived from WC and height through a geometrical elliptical model, provides a more accurate estimate of the VAT volume, as originally validated by Thomas et al. [8]. VAT is metabolically distinct from subcutaneous fat tissue: it is highly lipolytic, is in close anatomical proximity to the portal circulation, and secretes proinflammatory mediators, including tumor necrosis factor-α (TNF-α), interleukin-6 (IL-6), and monocyte chemoattractant protein-1 (MCP-1), while simultaneously reducing the levels of anti-inflammatory and insulin-sensitizing adiponectin [14,15]. These inflammatory alterations promote a systemic, protumorigenic environment characterized by chronic, low-grade inflammation, oxidative stress, and immune dysregulation [14].

The insulin/IGF-1 axis is a well-characterized mechanistic pathway that links visceral adiposity to colorectal carcinogenesis. Increased VAT is closely associated with hepatic insulin resistance, compensatory hyperinsulinemia, and elevated levels of circulating IGF-1 [15]. Insulin and IGF-1 directly stimulate proliferation and inhibit apoptosis in colorectal epithelial cells through activation of the PI3K/Akt and mitogen-activated protein kinase/extracellular signal-regulated kinase (MAPK/ERK) signaling pathways [14]. Experimental evidence further supports the involvement of this mechanistic pathway: diet-induced obesity in murine azoxymethane/dextran sulfate sodium (AOM/DSS) models significantly increases the incidence of colonic tumors and serum IGF-1 and leptin levels while expanding visceral fat, effects that can be reversed with caloric restriction [16]. These mechanistic data lend biological plausibility to the dose-response BRI–CRC association we observed in epidemiological settings.

Our findings align with and extend prior meta-analytic evidence. Moghaddam et al. pooled the data of 31 studies (70,000 CRC cases) and reported a relative risk of 1.45 (95% CI 1.31–1.61) for the highest (vs. lowest as the reference) category of central obesity (as measured by WC, whereas general obesity (BMI ≥ 30 kg/m^2^) yielded a relative risk of only 1.19 [17]. Our fully adjusted HR of 1.104 for the BRI Q4 vs. Q1 relative comparison is smaller in absolute magnitude, likely reflecting the additional adjustments for metabolic comorbidities and the fact that the BRI quartiles do not correspond to the extreme tails of the WC distribution used in the aforementioned meta-analysis. A dose-response meta-analysis of VAT and colorectal adenomas by Keum et al. revealed that each 25 cm^2^ increase in VAT area was associated with a 13% increase in adenoma risk (OR 1.13, 95% CI 1.05–1.21) and that these VAT associations persisted after adjustment for BMI and WC, which is consistent with our finding that the BRI, as a VAT surrogate, independently predicts the risk of CRC beyond conventional confounders [18].

### 4.2. Sex-Specific Patterns: Mechanistic Interpretation

The strikingly different associations between the BRI and CRC in men versus women—strongly positive versus negative, respectively—represent one of the most clinically significant findings of this study. This sex-specific pattern is consistent with and mechanistically explicable by fundamental differences in the distribution of adipose tissue between the sexes. In men, WC (a key component of the BRI) is strongly correlated with VAT volume, as men preferentially accumulate fat in the visceral compartment. In women, however, the abdominal wall contains a substantially greater proportion of subcutaneous adipose tissue (SAT) than VAT, such that a given increase in WC is more likely to reflect SAT expansion than VAT accumulation [19]. Compared with VAT, SAT is less active and does not drive the same degree of systemic insulin resistance, inflammation, or adipokine dysregulation [15]. Consequently, the BRI may systematically overestimate metabolically harmful visceral fat in women relative to men, resulting in an attenuation and reversal of the apparent BRI–CRC relationship observed in men.

Hormonal factors compound this anatomical explanation. In premenopausal women, endogenous estrogen exerts anticarcinogenic effects on the colorectal mucosa, increasing epithelial differentiation, reducing prostaglandin synthesis, and modulating bile acid metabolism, all of which may attenuate the carcinogenic influence of adiposity [7]. The higher BMIs and WC in women with higher BRI may, paradoxically, be associated with higher estrogen levels from peripheral aromatization in adipose tissue, providing partial protection against CRC independent of the metabolic harms of visceral fat. A prospective study by Song et al. revealed that the level of adiponectin was significantly inversely associated with the risk of CRC in men (highest vs. lowest quartile RR 0.55, 95% CI 0.35–0.86) but not in women (RR 0.96, 95% CI 0.67–1.39; *p* for heterogeneity < 0.05), suggesting that the operation of the adipokine-mediated pathways linking adiposity to CRC depend on sex [20]. The sex-specific patterns we observed for the BRI are therefore consistent with the findings of the epidemiological literature showing that central obesity is more strongly associated with CRC in men than in women [7,17].

### 4.3. Age-Dependent Associations

The absence of a significant BRI–CRC association in participants under 40 years, the presence of a strong association in the 40–64-year-old group, and the attenuation of this association in those aged ≥65 years suggest an age-dependent modification of the relationship between visceral adiposity and CRC. The lack of an association in young adults (<40 years) likely reflects the short duration of visceral fat exposure in this age group: the carcinogenic effects of hyperinsulinemia, chronic inflammation, and adipokine dysregulation on colorectal epithelial transformation require sustained visceral fat exposure over decades, which is consistent with the adenoma-to-carcinoma progression taking 10–15 years from initiation to clinically detectable cancer [21]. In this context, visceral fat accumulation beginning in early adulthood would be expected to have the greatest epidemiological effects in individuals aged 40–64 years, in whom such cumulative exposure has been substantial but in whom competing causes of death have not yet substantially depleted the at-risk population. The attenuation observed in those ≥65 years may reflect survivor bias (individuals with high BRI who developed metabolic complications or cardiovascular disease may have died before the potential manifestation of CRC), age-related changes in body composition that weaken the correlation between the BRI and true VAT, or greater contributions of nonmetabolic CRC risk factors (e.g., accumulated mucosal genetic damage, inflammatory bowel disease, or polypectomy history) in the oldest age group.

Because 11 prespecified subgroups were analyzed with both quartile-based and simplified parameterizations, we applied Benjamini–Hochberg false discovery rate correction to all interaction tests (Supplementary Table S3b), with corresponding hazard ratios for each interaction term detailed in Supplementary Tables S6 (main parameterization) and S7 (simplified parameterization). The strong sex, age, BMI, smoking, and alcohol interactions were preserved after FDR adjustment (all FDR-adjusted *p* < 0.001), and the diabetes interaction remained statistically significant (FDR-adjusted *p* = 0.048). By contrast, the borderline income interaction did not survive FDR correction in the main parameterization (FDR-adjusted *p* = 0.084), and we therefore treat this finding as hypothesis-generating rather than as a definitive basis for clinically actionable inference. Any subgroup-specific clinical framing in this manuscript therefore rests only on findings that are robust to FDR adjustment.

### 4.4. Interactions with the BMI and the Role of Combined Adiposity Assessments

The significant BMI × BRI interaction (*p* < 0.001), in which the BRI was associated with an increased risk of CRC only among those with a BMI ≥ 25 kg/m^2^ but not among those with a lower BMI, has important clinical implications. Among nonobese individuals according to Asian BMI criteria (BMI < 25 kg/m^2^), the highest BRI quartile corresponds to a WC and body shape configuration that is not necessarily pathological: individuals in this cohort whose BRI was in the first quartile had a mean BMI of 20.57 kg/m^2^, while the WC range of individuals whose BRI was in the fourth quartile and whose BMI was <25 kg/m^2^ may predominantly reflect lean body habitus with a proportionally normal WC rather than true visceral fat excess. Conversely, in the context of general obesity (BMI ≥ 25 kg/m^2^), a high BRI reliably identifies individuals with compounded VAT and metabolic dysfunction, for whom the carcinogenic pathways described above are most strongly activated. Although the quartile-based Q4 aHR in the BMI ≥ 25 kg/m^2^ subgroup did not reach statistical significance (aHR 1.212, 95% CI 0.996–1.474), the simplified Q4-vs.-Q1–Q3 analysis in this subgroup was statistically significant (aHR 1.097, 95% CI 1.065–1.131), and the formal BMI × BRI interaction ratio at Q4 was 1.228 (95% CI 1.007–1.498; Supplementary Table S6; corresponding simplified-parameterization interaction ratios are provided in Supplementary Table S7). Taken together, these findings suggest that the BRI provides additional discriminatory information for CRC risk in individuals with established general obesity, although the top-quartile point estimate should be interpreted with appropriate caution given its borderline statistical significance in the strict quartile-based subgroup analysis.

When BMI was added to Model 3 as a continuous covariate, the BRI–CRC association was substantially attenuated (Q4 aHR 0.983, 95% CI 0.947–1.020; Supplementary Table S8). This attenuation reflects statistical overadjustment rather than confounding, because BMI and BRI share a large fraction of variance (Pearson r ≈ 0.78) as both are derived from overlapping anthropometric measurements. When BMI was instead entered as a dichotomous obesity indicator (BMI ≥ 25 kg/m^2^), the Q4-vs.-Q1 association remained significant (aHR 1.051, 95% CI 1.018–1.085), consistent with an independent contribution of BRI to CRC risk beyond general obesity. This pattern parallels well-recognized cautions regarding multicollinearity among highly correlated anthropometric indices and reinforces the interpretation that BRI provides risk information complementary to, rather than redundant with, BMI.

### 4.5. Site-Specific Differences in BRI–CRC Associations

The gradient in the BRI associations across colorectal subsites—strongest for the proximal colon (Q4 aHR 1.363), intermediate for the distal colon (Q4 aHR 1.244), and weakest for the rectum (Q4 aHR 1.070)—aligns with established biological and epidemiological evidence. Proximal and distal colorectal tumors differ in embryological origin (midgut vs. hindgut), gene expression profile, somatic mutation patterns, and microenvironmental characteristics. The proximal colon is particularly enriched in bile acid-metabolizing bacteria and experiences greater exposure to secondary bile acids, whose abundances are increased by visceral fat–related dyslipidemia and hyperinsulinemia and promote colonic epithelial proliferation and DNA damage [21]. Furthermore, cardiovascular risk factors—which are closely associated with visceral adiposity—have been shown to be more strongly associated with proximal CRC than with distal or rectal cancers [22]. The relatively weaker association between BRI and rectal cancer may also reflect anatomical distinctness of the rectal compartment, whose mesorectal fat depot and lymphatic drainage differ substantially from those of the colon proper [17]. Although direct mesorectal fat imaging was not available in the present cohort, so any anatomical explanation must remain hypothesis-generating, emerging techniques such as fluorescence-guided mesorectal nodal assessment demonstrate that precise, imaging-based mesorectal characterization is technically feasible and could enable future studies to directly quantify the contribution of local visceral fat to rectal carcinogenesis [23].

### 4.6. Strengths and Limitations

This study has several notable strengths. The nationwide, population-based design, which encompassed more than 4.4 million individuals, provides exceptional statistical power, allowing the detection of modest effect sizes and robust estimations of subgroup-specific associations across 11 prespecified variables. The linkage to the NHIS cancer registry, which mandates reporting of all incident cancers by treating physicians and links registration to copayment reduction benefits, provides the determination of CRC outcomes with high sensitivity and specificity and minimal risk of detection bias. The long follow-up duration (mean 10.32 years) and incorporation of standardized, government-administered anthropometric measurements minimize measurement error and ensure high data quality.

Several limitations must also be acknowledged. First, the BRI was assessed at a single baseline examination. Because the Korean NHIS is a claim-based national administrative database in which biennial health check-ups are performed by numerous certified examiners across thousands of institutions, it is optimized for detecting population-level associations rather than individual longitudinal phenotype trajectories. Consequently, time-varying exposure models and BRI trajectory clustering, as recently applied by Chen et al. in a purpose-designed longitudinal cohort, were not feasible in this database [24]. This structural constraint should be understood as inherent to the data source rather than a discretionary design choice. Future studies within cohorts specifically designed for serial anthropometric assessment will be needed to characterize the influence of intra-individual BRI change on CRC risk. Future studies incorporating serial BRI measurements from multiple health examinations could better delineate the role of the long-term BRI trajectory in the risk of CRC, building on the trajectory analysis framework recently applied to the BRI and overall cancer risk by Chen et al. [24]. Second, the NHIS database does not include data on dietary habits (e.g., red and processed meat intake, dietary fiber, and alcohol consumption quantity beyond the categorical variable), physical activity beyond a binary regular exercise indicator, family history of CRC, colonoscopy and polypectomy history, use of aspirin or NSAIDs, and menopausal status in women, all of which are established CRC risk modifiers that could confound the observed associations. Third, despite the imposition of a 1-year lag period and comprehensive adjustments for covariates, residual confounding from unmeasured variables cannot be excluded in this observational design. Fourth, the unexpected inverse association between the BRI and CRC in women requires careful interpretation and further investigation with direct imaging-based visceral fat measurements (e.g., CT-based VAT quantification) rather than the use of an anthropometric surrogate as provided by the BRI. Fifth, although our cohort was limited to Korean adults, the NHIS 2012 data represent a 40% random sample of the total Korean population aged ≥20 years, providing high internal validity, whereas generalizability to other Asian populations or to Western populations with different body composition norms should be evaluated in separate studies. Participants with missing baseline data were excluded rather than imputed; although this proportion (5.9%) is modest and consistent with prior NHIS-based literature, residual selection bias from non-random missingness cannot be entirely excluded.

To quantitatively address the potential impact of unmeasured confounders that are not captured in the Korean NHIS health check-up questionnaire—including dietary patterns, screening colonoscopy and polypectomy history, NSAID use, menopausal status in women, and family history of CRC—we calculated E-values (Supplementary Table S5). The E-value for the overall Q4-vs.-Q1 association was 1.44 (lower CI limit 1.36), meaning that an unmeasured confounder would have to be associated with both the BRI and CRC by a risk ratio of at least 1.44, above and beyond the measured covariates, to fully explain away the observed association. For the male-specific Q4 estimate, the corresponding E-values were substantially larger (2.04 for the point estimate; 1.93 for the CI limit), suggesting that the male-specific association is robust to plausible unmeasured confounding of the magnitude typical of dietary and lifestyle factors in observational CRC epidemiology. The weakest robustness was observed for rectal cancer (E-value CI limit 1.12), consistent with the smaller effect size at that anatomical site.

To confirm that the reported sex interaction was not an artifact of overall quartile construction, we repeated the analysis using sex-stratified BRI quartiles (Supplementary Table S9a,b. The Q4-vs.-Q1 aHR under sex-stratified quartiles (1.127, 95% CI 1.097–1.158) was quantitatively similar to that from the pooled quartile analysis (1.104, 95% CI 1.075–1.135), and the qualitative sex-interaction pattern was preserved, indicating that our sex-modification findings are robust to quartile-construction choices.

## 5. Conclusions

In this nationwide prospective cohort study of 4,492,697 Korean adults followed for a mean of 10.32 years, a higher BRI was independently and dose-dependently associated with an increased risk of CRC, with a fully adjusted hazard ratio of 1.104 (95% CI 1.075–1.135) for the highest versus the lowest BRI quartile (*p* for trend < 0.001). Subgroup analyses revealed that this association was strongest in men (Q4 aHR 1.352) but paradoxically inverse in women (Q4 aHR 0.891) among middle-aged adults (40–64 years) and in those with general obesity (BMI ≥ 25 kg/m^2^). Across specific colorectal subsites, the BRI–CRC association followed a proximal-to-distal gradient, with the strongest association for proximal colon cancer (Q4 aHR 1.363) and the lowest for rectal cancer (Q4 aHR 1.070). These findings are mechanistically consistent with the established roles of visceral adiposity-driven hyperinsulinemia, chronic inflammation, and adipokine dysregulation in colorectal carcinogenesis and are supported by increasing biological evidence from experimental models and prior epidemiological meta-analyses.

The BRI, derived from two routine measurements—WC and height—is a practical and readily implementable anthropometric index for estimating visceral fat in primary health care and national health examination settings. Our results suggest that the BRI should be considered a complementary measure alongside the BMI when stratifying the risk of CRC, particularly among men and obese individuals, for whom its discriminatory value appears greatest. Population-level interventions that target reductions in visceral fat and prospective studies validating BRI-based risk stratification tools are warranted to translate these epidemiological findings into clinical and public health practice.

## Figures and Tables

**Table 1:** Baseline characteristics of the study participants stratified by body roundness index (BRI) quartile.

Characteristic	Total (n = 4,492,697)	Q1 (n = 1,125,142)	Q2 (n = 1,122,850)	Q3 (n = 1,121,668)	Q4 (n = 1,123,037)
BRI range	—	≤2.48	2.49–3.12	3.13–3.82	≥3.83
**Age, years (mean ± SD)**					
20–39 years, n (%)	1,257,173 (27.98)	556,009 (49.42)	330,044 (29.39)	222,397 (19.83)	148,723 (13.24)
40–64 years, n (%)	2,661,730 (59.25)	527,935 (46.92)	706,199 (62.89)	749,542 (66.82)	678,054 (60.38)
≥65 years, n (%)	573,794 (12.77)	41,198 (3.66)	86,607 (7.71)	149,729 (13.35)	296,260 (26.38)
Male sex, n (%)	2,441,428 (54.34)	471,444 (41.90)	665,579 (59.28)	726,994 (64.81)	577,411 (51.42)
Low income/MAB, n (%)	838,757 (18.67)	207,736 (18.46)	195,871 (17.44)	200,545 (17.88)	234,605 (20.89)
**Anthropometric characteristics**					
BMI, kg/m^2^ (mean ± SD)	23.77 ± 3.28	20.57 ± 1.91	22.91 ± 1.91	24.55 ± 2.10	27.05 ± 3.01
<18.5, n (%)	163,239 (3.63)	148,679 (13.21)	10,515 (0.94)	2714 (0.24)	1331 (0.12)
18.5–22.9, n (%)	1,737,023 (38.66)	859,432 (76.38)	570,139 (50.78)	240,555 (21.45)	66,897 (5.96)
23.0–24.9, n (%)	1,097,871 (24.44)	101,752 (9.04)	388,726 (34.62)	412,865 (36.81)	194,528 (17.32)
25.0–29.9, n (%)	1,316,592 (29.31)	14,991 (1.33)	152,216 (13.56)	455,585 (40.62)	693,800 (61.78)
≥30.0, n (%)	177,972 (3.96)	288 (0.03)	1254 (0.11)	9949 (0.89)	166,481 (14.82)
WC, cm (mean ± SD)	80.29 ± 9.28	69.72 ± 5.20	77.79 ± 4.55	83.24 ± 4.72	90.43 ± 6.70
Abdominal obesity, n (%)	901,966 (20.08)	2 (0.00)	2564 (0.23)	114,784 (10.23)	784,616 (69.87)
**Laboratory values**					
SBP, mmHg (mean ± SD)	121.96 ± 14.79	115.13 ± 13.08	120.58 ± 13.76	124.09 ± 14.14	128.04 ± 14.97
DBP, mmHg (mean ± SD)	76.11 ± 9.98	72.25 ± 9.15	75.45 ± 9.53	77.49 ± 9.74	79.25 ± 10.09
Fasting glucose, mg/dL	97.75 ± 23.23	91.16 ± 16.33	95.90 ± 20.91	99.78 ± 24.13	104.20 ± 27.92
Total cholesterol, mg/dL	194.79 ± 36.62	184.10 ± 32.67	194.48 ± 35.21	199.15 ± 36.83	201.46 ± 39.02
HDL cholesterol, mg/dL	55.50 ± 17.56	61.53 ± 19.72	55.92 ± 17.28	52.93 ± 16.09	51.62 ± 15.11
LDL cholesterol, mg/dL	113.96 ± 33.96	104.59 ± 29.66	114.39 ± 32.62	117.87 ± 34.69	119.00 ± 36.57
Triglycerides †, mg/dL	109.6 (109.6–109.7)	80.0 (80.0–80.1)	105.8 (105.7–105.9)	124.7 (124.6–124.8)	137.0 (136.8–137.1)
eGFR, mL/min/1.73 m^2^	91.96 ± 35.95	95.78 ± 35.29	92.73 ± 37.06	90.72 ± 36.73	88.59 ± 34.24
**Comorbidities**					
Diabetes mellitus, n (%)	283,697 (6.31)	20,607 (1.83)	49,348 (4.39)	82,756 (7.38)	130,986 (11.66)
Hypertension, n (%)	619,013 (13.78)	53,643 (4.77)	114,134 (10.16)	177,009 (15.78)	274,227 (24.42)
Hypercholesterolemia, n (%)	498,097 (11.09)	61,246 (5.44)	113,182 (10.08)	147,996 (13.19)	175,673 (15.64)
CKD (eGFR <60), n (%)	166,273 (3.70)	18,713 (1.66)	28,741 (2.56)	42,527 (3.79)	76,292 (6.79)
**Lifestyle variables**					
Never smoker, n (%)	2,674,063 (59.52)	756,205 (67.21)	630,273 (56.13)	589,505 (52.56)	698,080 (62.16)
Former smoker, n (%)	698,835 (15.55)	107,251 (9.53)	180,234 (16.05)	222,503 (19.84)	188,847 (16.82)
Current smoker, n (%)	1,119,799 (24.92)	261,686 (23.26)	312,343 (27.82)	309,660 (27.61)	236,110 (21.02)
No alcohol consumption, n (%)	2,270,578 (50.54)	564,596 (50.18)	527,861 (47.01)	529,179 (47.18)	648,942 (57.78)
Mild alcohol consumption, n (%)	1,860,774 (41.42)	492,018 (43.73)	502,123 (44.72)	487,076 (43.42)	379,557 (33.80)
Heavy alcohol consumption, n (%)	361,345 (8.04)	68,528 (6.09)	92,866 (8.27)	105,413 (9.40)	94,538 (8.42)
Participation in regular exercise, n (%)	853,531 (19.00)	192,813 (17.14)	227,779 (20.29)	229,223 (20.44)	203,716 (18.14)

All *p* values < 0.001 across quartile groups. Abbreviations: WC, waist circumference; SBP/DBP, systolic/diastolic blood pressure; HDL/LDL, high/low-density lipoprotein; eGFR, estimated glomerular filtration rate; CKD, chronic kidney disease; MAB, medical aid beneficiary. † Triglyceride levels are expressed as the geometric mean (95% CI).

**Table 2:** Hazard ratios (HRs) and 95% confidence intervals (CIs) for incident colorectal cancer according to body roundness index (BRI) quartile.

BRI	N	Events	Person-Years	Incidence/1000 py	Model 1 HR (95% CI)	Model 2 HR (95% CI)	Model 3 HR (95% CI)
Q1	1,125,142	8430	11,625,321	0.725	1 (Ref.)	1 (Ref.)	1 (Ref.)
Q2	1,122,850	12,302	11,592,825	1.061	1.463 (1.423–1.505)	1.044 (1.015–1.074)	1.030 (1.002–1.059)
Q3	1,121,668	15,960	11,572,086	1.379	1.902 (1.853–1.953)	1.093 (1.064–1.123)	1.067 (1.038–1.097)
Q4	1,123,037	20,952	11,592,455	1.807	2.492 (2.430–2.556)	1.147 (1.116–1.178)	1.104 (1.075–1.135)
***p* for trend**	—	—	—	—	**<0.001**	**<0.001**	**<0.001**

Abbreviations: py: person-years; Ref.: reference. Model 1: unadjusted. Model 2: Model 1 adjusted for age and sex. Model 3: Model 2 additionally adjusted for smoking, alcohol, participation in regular exercise, income, diabetes mellitus status, hypertension status, hypercholesterolemia status, and chronic kidney disease status.

**Table 3:** Selected subgroup analyses of BRI quartiles and colorectal cancer risk (age, sex, BMI, diabetes status).

Subgroup	BRI	N	Events	Person-Years	Incidence/1000 py	aHR (95% CI)	*p* for Interaction
Age group							<0.001
<40 years	Q1	556,009	1711	5,750,022	0.298	1 (Ref.)	
	Q2	330,044	1041	3,409,219	0.305	0.866 (0.802–0.935)	
	Q3	222,397	772	2,294,193	0.337	0.898 (0.824–0.978)	
	Q4	148,723	558	1,531,897	0.364	0.970 (0.881–1.067)	
40–64 years	Q1	527,935	5487	5,448,924	1.007	1 (Ref.)	
	Q2	706,199	8447	7,287,584	1.159	1.000 (0.966–1.034)	
	Q3	749,542	10,286	7,729,191	1.331	1.031 (0.997–1.066)	
	Q4	678,054	10,827	6,991,228	1.549	1.126 (1.089–1.164)	
≥65 years	Q1	41,198	1232	426,376	2.889	1 (Ref.)	
	Q2	86,607	2814	896,022	3.141	1.125 (1.052–1.203)	
	Q3	149,729	4902	1,548,701	3.165	1.137 (1.068–1.211)	
	Q4	296,260	9567	3,069,331	3.117	1.125 (1.060–1.194)	
Sex							<0.001
Male	Q1	471,444	3779	4,873,713	0.775	1 (Ref.)	
	Q2	665,579	7332	6,867,674	1.068	1.123 (1.080–1.168)	
	Q3	726,994	10,736	7,488,769	1.434	1.231 (1.186–1.278)	
	Q4	577,411	11,396	5,938,484	1.919	1.352 (1.302–1.404)	
Female	Q1	653,698	4651	6,751,608	0.689	1 (Ref.)	
	Q2	457,271	4970	4,725,151	1.052	0.974 (0.936–1.014)	
	Q3	394,674	5224	4,083,317	1.279	0.915 (0.879–0.953)	
	Q4	545,626	9556	5,653,971	1.690	0.891 (0.858–0.925)	
BMI							<0.001
<25 kg/m^2^	Q1	1,109,863	8329	11,467,585	0.726	1 (Ref.)	
	Q2	969,380	11,165	10,010,523	1.115	1.035 (1.005–1.065)	
	Q3	656,134	10,866	6,774,348	1.604	1.064 (1.033–1.096)	
	Q4	262,756	5813	2,719,974	2.137	0.986 (0.952–1.023)	
≥25 kg/m^2^	Q1	15,279	101	157,736	0.640	1 (Ref.)	
	Q2	153,470	1137	1,582,302	0.719	1.020 (0.832–1.250)	
	Q3	465,534	5094	4,797,737	1.062	1.122 (0.921–1.366)	
	Q4	860,281	15,139	8,872,480	1.706	1.212 (0.996–1.474)	
Diabetes mellitus							0.026
No	Q1	1,104,535	8096	11,413,063	0.709	1 (Ref.)	
	Q2	1,073,502	11,311	11,085,414	1.020	1.025 (0.995–1.055)	
	Q3	1,038,912	14,067	10,721,909	1.312	1.060 (1.031–1.090)	
	Q4	992,051	17,692	10,244,422	1.727	1.109 (1.078–1.140)	
Yes	Q1	20,607	334	212,258	1.574	1 (Ref.)	
	Q2	49,348	991	507,411	1.953	1.125 (0.994–1.274)	
	Q3	82,756	1893	850,176	2.227	1.160 (1.033–1.303)	
	Q4	130,986	3260	1,348,033	2.418	1.122 (1.002–1.255)	

Full subgroup results for all 11 covariates are provided in Supplementary Table S4. aHR: adjusted hazard ratio; py: person-years; Ref.: reference. All the models were adjusted for age, sex, smoking status, alcohol consumption, participation in regular exercise, income, diabetes status, hypertension status, hypercholesterolemia status and chronic kidney disease status (except for the stratifying variable). *p* for interaction computed using multiplicative interaction terms.

## Data Availability

Data are available from the corresponding authors upon reasonable request with the permission of the Korean National Health Insurance Service, as restrictions apply to these data, which were used under license for this study.

## Supplementary Materials

The following supporting information can be downloaded at: https://www.mdpi.com/article/10.3390/cancers18152466/s1, Table S1. Number at Risk by BRI Quartile at Annual Intervals (Corresponding to Figure 2). Table S2. Site-Specific Colorectal Cancer Risk by BRI Quartile. Table S3a. Full Subgroup Analysis—Q1 through Q4 across All Prespecified Covariates. Table S3b. Subgroup interaction P values, main (quartile-based) parameterization, with Benjamini–Hochberg FDR adjustment. Table S4. Simplified Subgroup Analysis—Q4 vs. Q1–Q3. Table S5. E-values for the principal BRI–CRC associations. The E-value represents the minimum strength of association, on the risk ratio scale, that an unmeasured confounder would need to have with both the exposure (BRI) and the outcome (CRC), above and beyond the measured covariates, to fully explain away the observed association. Table S6. Ratios of hazard ratios for each interaction variable across BRI quartiles Q2, Q3, and Q4 relative to Q1, from the main quartile-based parameterization of Model 3. Table S7. Ratios of hazard ratios for each interaction variable, from the simplified Q4-vs-Q1–Q3 parameterization of Model 3. Table S8. Sensitivity analyses: extended lag periods, restricted follow-up, Fine–Gray subdistribution hazard model, and alternate BMI adjustment specifications. Table S9a. Sex-specific BRI quartile cutoffs. Table S9b. BRI and incident CRC risk using sex-stratified quartiles (Model 3, fully adjusted).

## Abbreviations

The following abbreviations are used in this manuscript:

CRC	colorectal cancer
BRI	body roundness index
BMI	body mass index
HR	hazard ratio
CI	confidence interval
IGF	insulin-like growth factor
WC	waist circumference
NHANES	National Health and Nutrition Examination Survey
NHIS	National Health Insurance Service
IR	incidence rate
PY	person-years
CKD	chronic kidney disease
eGFR	estimated glomerular filtration rate
MDRD	Modification of Diet in Renal Disease
SD	standard deviation
ANOVA	analysis of variance
SBP	systolic blood pressure
DBP	diastolic blood pressure
HDL	high-density lipoprotein
LDL	low-density lipoprotein
MAB	medical aid beneficiary
Ref.	reference
aHR	adjusted hazard ratio
VAT	visceral adipose tissue
TNF-α	tumor necrosis factor-α
IL-6	interleukin-6
MCP-1	monocyte chemoattractant protein-1
MAPK	mitogen-activated protein kinase
ERK	extracellular signal-regulated kinase
AOM	azoxymethane
DSS	dextran sulfate sodium
SAT	subcutaneous adipose tissue
