# Supplementary material for: Body Roundness Index and Incident Colorectal Cancer Risk: A Nationwide Population-Based Cohort Study
Source: Cancers (Basel). 2026 Jul 31;18(15):2466. doi: 10.3390/cancers18152466 (PMC13464952; doi:10.3390/cancers18152466)
Supplement: Supplementary file 1 [file cancers-18-02466-s001.zip › cancers-4452938-supplementary.pdf]

Supplementary Material

Body Roundness Index and Incident Colorectal Cancer Risk: A Nationwide Population-Based Cohort Study

Supplementary Table S1. Number at Risk by BRI Quartile at Annual Intervals (Corresponding to Figure 2).

Supplementary Table S1 presents the number of participants at risk at each annual time point by BRI quartile, corresponding to Figure 2 in the main manuscript. Overall log-rank P <0.0001; all six pairwise between-quartile comparisons yielded Bonferroni-adjusted P <0.0001.

BRI group	0	1	2	3	4	5	6	7	8	9	10
**Q1**	1,125,142	1,124,538	1,123,845	1,123,145	1,122,404	1,121,667	1,120,937	1,120,134	1,119,119	1,118,128	1,099,181
**Q2**	1,122,850	1,121,880	1,120,818	1,119,743	1,118,654	1,117,604	1,116,451	1,115,305	1,113,929	1,112,572	1,093,291
**Q3**	1,121,668	1,120,330	1,118,888	1,117,441	1,115,942	1,114,461	1,112,849	1,111,369	1,109,684	1,108,059	1,089,157
**Q4**	1,123,037	1,121,257	1,119,288	1,117,257	1,115,269	1,113,333	1,111,219	1,109,309	1,107,218	1,105,066	1,086,908

Number of participants at risk by body roundness index (BRI) quartile at annual intervals, corresponding to Figure 2. BRI quartiles: Q1 ≤2.48; Q2 2.49–3.12; Q3 3.13–3.82; Q4 ≥3.83. Overall log-rank P <0.0001.

Supplementary Table S2. Site-Specific Colorectal Cancer Risk by BRI Quartile.

Supplementary Table S2 presents fully adjusted hazard ratios for site-specific colorectal cancer (proximal colon, distal colon, and rectum) by BRI quartile. The association was strongest for proximal colon cancer (Q4 aHR 1.363, 95% CI 1.254–1.481) and weakened progressively towards the rectum (Q4 aHR 1.070, 95% CI 1.011–1.132).

BRI	N	Person-years	Proximal colon cancer (C18.0–18.4)			Distal colon cancer (C18.5–18.7, C19.0)			Rectal cancer (C20.0)		
			Events	Incidence/1000 py	aHR (95% CI)	Events	IR/1000py	aHR (95% CI)	Events	Incidence/1000 py	aHR (95% CI)
Q1	1,125,142	11,625,321	791	0.068	1 (Ref.)	1,336	0.115	1 (Ref.)	2,059	0.177	1 (Ref.)
Q2	1,122,850	11,592,825	1,368	0.118	1.161 (1.063–1.268)	2,099	0.181	1.027 (0.959–1.101)	2,960	0.255	1.029 (0.972–1.090)

Q3	1,121,668	11,572,086	1,917	0.166	1.240 (1.139–1.349)	3,080	0.266	1.150 (1.077–1.229)	3,716	0.321	1.056 (0.998–1.116)
Q4	1,123,037	11,592,455	3,004	0.259	1.363 (1.254–1.481)	4,193	0.362	1.244 (1.166–1.328)	4,353	0.376	1.070 (1.011–1.132)
**P trend**				**<0.001**			**<0.001**			**<0.001**	

Site-specific colorectal cancer risk by BRI quartile. aHR: adjusted hazard ratio (Model 3: age, sex, smoking status, alcohol consumption, participation in regular exercise, income, diabetes status, hypertension status, hypercholesterolemia status, CKD status). py: person-years; Ref.: reference. Unspecified (C18.9) and overlapping (C18.8) lesions were excluded from site-specific analyses.

Supplementary Table S3a. Full Subgroup Analysis — Q1 through Q4 across All Prespecified Covariates.

Supplementary Table S3a provides complete subgroup analyses for all 11 prespecified covariates not presented in Table 3 of the main manuscript (income, smoking status, alcohol consumption, participation in regular exercise, hypertension status, hypercholesterolemia status, and CKD status), along with all the results for variables also shown in Table 3 (age, sex, BMI, and diabetes status). Each subgroup is stratified by BRI quartile (Q1–Q4). FDR-adjusted P values for all subgroup interactions are provided in Supplementary Table S3b.

Subgroup	BRI	N	Events	Person-years	Incidence /1000 py	aHR (95% CI)	*p* for interaction
Income							0.054
High (Q2–4)	Q1	917,406	6,675	9,481,782	0.704	1 (Ref.)	
	Q2	926,979	9,745	9,572,961	1.018	1.022 (0.991–1.055)	
	Q3	921,123	12,536	9,505,111	1.319	1.058 (1.026–1.091)	
	Q4	888,432	16,265	9,171,808	1.773	1.110 (1.077–1.144)	
Low/MAB	Q1	207,736	1,755	2,143,539	0.819	1 (Ref.)	
	Q2	195,871	2,557	2,019,864	1.266	1.061 (0.998–1.128)	
	Q3	200,545	3,424	2,066,975	1.657	1.104 (1.042–1.170)	
	Q4	234,605	4,687	2,420,646	1.936	1.088 (1.029–1.150)	
Smoking status							<0.001
Never	Q1	756,205	5,274	7,814,711	0.675	1 (Ref.)	
	Q2	630,273	6,554	6,513,747	1.006	0.991 (0.955–1.028)	
	Q3	589,505	7,961	6,093,545	1.306	0.993 (0.958–1.030)	
	Q4	698,080	12,599	7,224,598	1.744	0.993 (0.960–1.028)	
Former	Q1	107,251	999	1,108,647	0.901	1 (Ref.)	
	Q2	180,234	2,290	1,860,270	1.231	1.117 (1.037–1.204)	
	Q3	222,503	3,628	2,292,476	1.583	1.183 (1.103–1.270)	
	Q4	188,847	4,283	1,941,136	2.206	1.335 (1.246–	

						1.431)	
Current	Q1	261,686	2,157	2,701,964	0.798	1 (Ref.)	
	Q2	312,343	3,458	3,218,808	1.074	1.097 (1.040–1.158)	
	Q3	309,660	4,371	3,186,065	1.372	1.189 (1.129–1.252)	
	Q4	236,110	4,070	2,426,721	1.677	1.258 (1.194–1.326)	
Alcohol consumption							<0.001
None	Q1	564,596	4,566	5,833,662	0.783	1 (Ref.)	
	Q2	527,861	6,193	5,454,984	1.135	1.003 (0.965–1.042)	
	Q3	529,179	7,990	5,469,450	1.461	1.021 (0.984–1.060)	
	Q4	648,942	12,602	6,715,690	1.877	1.019 (0.984–1.056)	
Mild (<30 g/day)	Q1	492,018	3,251	5,083,924	0.639	1 (Ref.)	
	Q2	502,123	4,992	5,180,245	0.964	1.074 (1.028–1.123)	
	Q3	487,076	6,320	5,016,954	1.260	1.123 (1.076–1.172)	
	Q4	379,557	6,525	3,904,355	1.671	1.234 (1.182–1.289)	
Heavy (≥30 g/day)	Q1	68,528	613	707,736	0.866	1 (Ref.)	
	Q2	92,866	1,117	957,595	1.166	1.024 (0.928–1.130)	
	Q3	105,413	1,650	1,085,682	1.520	1.117 (1.018–1.226)	
	Q4	94,538	1,825	972,410	1.877	1.184 (1.080–1.298)	
Participation in regular exercise							0.420
No	Q1	932,329	6,812	9,630,077	0.707	1 (Ref.)	
	Q2	895,071	9,549	9,239,106	1.034	1.022 (0.990–1.054)	
	Q3	892,445	12,367	9,206,133	1.343	1.057 (1.026–1.090)	
	Q4	919,321	16,930	9,490,600	1.784	1.093 (1.060–1.126)	
Yes	Q1	192,813	1,618	1,995,244	0.811	1 (Ref.)	
	Q2	227,779	2,753	2,353,719	1.170	1.066 (1.002–1.134)	
	Q3	229,223	3,593	2,365,953	1.519	1.107 (1.044–1.175)	

	Q4	203,716	4,022	2,101,855	1.914	1.153 (1.088–1.223)	
Hypertension							0.671
No	Q1	1,071,499	7,701	11,072,573	0.696	1 (Ref.)	
	Q2	1,008,716	10,449	10,417,567	1.003	1.026 (0.996–1.057)	
	Q3	944,659	12,664	9,749,700	1.299	1.066 (1.035–1.097)	
	Q4	848,810	14,718	8,765,729	1.679	1.097 (1.066–1.130)	
Yes	Q1	53,643	729	552,748	1.319	1 (Ref.)	
	Q2	114,134	1,853	1,175,258	1.577	1.066 (0.979–1.162)	
	Q3	177,009	3,296	1,822,386	1.809	1.094 (1.009–1.185)	
	Q4	274,227	6,234	2,826,726	2.205	1.149 (1.064–1.240)	
Hypercholesterolemia							0.176
No	Q1	1,063,896	7,802	10,993,961	0.710	1 (Ref.)	
	Q2	1,009,668	10,926	10,426,017	1.048	1.031 (1.001–1.062)	
	Q3	973,672	13,940	10,046,904	1.387	1.075 (1.045–1.106)	
	Q4	947,364	17,930	9,779,241	1.833	1.112 (1.081–1.144)	
Yes	Q1	61,246	628	631,360	0.995	1 (Ref.)	
	Q2	113,182	1,376	1,166,808	1.179	1.006 (0.915–1.105)	
	Q3	147,996	2,020	1,525,181	1.324	0.992 (0.907–1.085)	
	Q4	175,673	3,022	1,813,214	1.667	1.032 (0.947–1.125)	
Chronic kidney disease							0.142
No	Q1	1,106,429	8,136	11,432,295	0.712	1 (Ref.)	
	Q2	1,094,109	11,748	11,296,171	1.040	1.033 (1.004–1.063)	
	Q3	1,079,141	14,998	11,132,818	1.347	1.072 (1.043–1.103)	
	Q4	1,046,745	18,841	10,803,877	1.744	1.112 (1.082–1.144)	
Yes	Q1	18,713	294	193,026	1.523	1 (Ref.)	
	Q2	28,741	554	296,654	1.867	0.941 (0.817–1.084)	
	Q3	42,527	962	439,268	2.190	0.935 (0.821–	

						1.066)	
	Q4	76,292	2,111	788,578	2.677	0.967 (0.856–1.093)	

Full subgroup analysis of BRI quartile and incident colorectal cancer risk across all 11 prespecified covariates. aHR: adjusted hazard ratio; py: person-years; Ref.: reference; MAB: medical aid beneficiary; CKD: chronic kidney disease. The model 3 was adjusted for age, sex, smoking status, alcohol consumption, participation in regular exercise, income, diabetes mellitus status, hypertension status, hypercholesterolemia status and chronic kidney disease status (except for the stratifying variable). P for interaction computed using multiplicative interaction terms.

Supplementary Table S3b. Subgroup interaction P values, main (quartile-based) parameterization, with Benjamini–Hochberg FDR adjustment.

Subgroup variable	Raw P (interaction)	FDR-adjusted P
Age group	<0.001	<0.001
Sex	<0.001	<0.001
BMI category	<0.001	<0.001
Smoking	<0.001	<0.001
Alcohol consumption	<0.001	<0.001
Diabetes	0.026	0.048
Income	0.054	0.084
Hypercholesterolemia	0.176	0.216
Chronic kidney disease	0.142	0.195
Regular exercise	0.420	0.462
Hypertension	0.671	0.671

Supplementary Table S4. Simplified Subgroup Analysis — Q4 vs. Q1–Q3.

Supplementary Table S4 presents a simplified subgroup analysis comparing participants in the highest BRI quartile (Q4) with those in the lower three quartiles combined (Q1–Q3) across all 11 prespecified covariates.

Subgroup	BRI	N	Events	Person-years	Incidence/1000 py	aHR (95% CI)	*p* for interaction
Age group							<0.001
<40 years	Q1–Q3	1,108,450	3,524	11,453,434	0.308	1 (Ref.)	
	Q4	148,723	558	1,531,897	0.364	1.038 (0.949–1.135)	
40–64 years	Q1–Q3	1,983,676	24,220	20,465,699	1.183	1 (Ref.)	
	Q4	678,054	10,827	6,991,228	1.549	1.111 (1.086–1.137)	
≥65 years	Q1–Q3	277,534	8,948	2,871,099	3.117	1 (Ref.)	
	Q4	296,260	9,567	3,069,331	3.117	1.011 (0.982–1.041)	
Sex (P for interaction <0.001)							

Male	Q1–Q3	1,864,017	21,847	19,230,156	1.136	1 (Ref.)	
	Q4	577,411	11,396	5,938,484	1.919	1.175 (1.148–1.202)	
Female	Q1–Q3	1,505,643	14,845	15,560,076	0.954	1 (Ref.)	
	Q4	545,626	9,556	5,653,971	1.690	0.926 (0.902–0.951)	
Income							0.016
High (Q2–4)	Q1–Q3	2,765,508	28,956	28,559,854	1.014	1 (Ref.)	
	Q4	888,432	16,265	9,171,808	1.773	1.072 (1.051–1.094)	
Low/MAB	Q1–Q3	604,152	7,736	6,230,377	1.242	1 (Ref.)	
	Q4	234,605	4,687	2,420,646	1.936	1.020 (0.983–1.058)	
Smoking (P for interaction <0.001)							
Never	Q1–Q3	1,975,983	19,789	20,422,002	0.969	1 (Ref.)	
	Q4	698,080	12,599	7,224,598	1.744	0.996 (0.973–1.020)	
Former	Q1–Q3	509,988	6,917	5,261,393	1.315	1 (Ref.)	
	Q4	188,847	4,283	1,941,136	2.206	1.178 (1.134–1.224)	
Current	Q1–Q3	883,689	9,986	9,106,836	1.097	1 (Ref.)	
	Q4	236,110	4,070	2,426,721	1.677	1.130 (1.089–1.172)	
Alcohol consumption							<0.001
None	Q1–Q3	1,621,636	18,749	16,758,096	1.119	1 (Ref.)	
	Q4	648,942	12,602	6,715,690	1.877	1.006 (0.983–1.030)	
Mild	Q1–Q3	1,481,217	14,563	15,281,122	0.953	1 (Ref.)	
	Q4	379,557	6,525	3,904,355	1.671	1.143 (1.109–1.177)	
Heavy	Q1–Q3	266,807	3,380	2,751,013	1.229	1 (Ref.)	
	Q4	94,538	1,825	972,410	1.877	1.111 (1.049–1.176)	
Participation in regular exercise (P for interaction = 0.406)							
No	Q1–Q3	2,719,845	28,728	28,075,316	1.023	1 (Ref.)	
	Q4	919,321	16,930	9,490,600	1.784	1.056 (1.035–1.078)	
Yes	Q1–Q3	649,815	7,964	6,714,916	1.186	1 (Ref.)	

	Q4	203,716	4,022	2,101,855	1.914	1.075 (1.035–1.117)	
BMI							<0.001
<25 kg/m²	Q1–Q3	2,735,377	30,360	28,252,456	1.075	1 (Ref.)	
	Q4	262,756	5,813	2,719,974	2.137	0.948 (0.921–0.976)	
≥25 kg/m²	Q1–Q3	634,283	6,332	6,537,775	0.969	1 (Ref.)	
	Q4	860,281	15,139	8,872,480	1.706	1.097 (1.065–1.131)	
Diabetes mellitus							0.004
No	Q1–Q3	3,216,949	33,474	33,220,386	1.008	1 (Ref.)	
	Q4	992,051	17,692	10,244,422	1.727	1.071 (1.050–1.091)	
Yes	Q1–Q3	152,711	3,218	1,569,845	2.050	1 (Ref.)	
	Q4	130,986	3,260	1,348,033	2.418	0.991 (0.944–1.041)	
Hypertension (P for interaction = 0.562)							
No	Q1–Q3	3,024,874	30,814	31,239,840	0.986	1 (Ref.)	
	Q4	848,810	14,718	8,765,729	1.679	1.057 (1.036–1.079)	
Yes	Q1–Q3	344,786	5,878	3,550,392	1.656	1 (Ref.)	
	Q4	274,227	6,234	2,826,726	2.205	1.070 (1.032–1.109)	
Hypercholesterolemia							0.232
No	Q1–Q3	3,047,236	32,668	31,466,882	1.038	1 (Ref.)	
	Q4	947,364	17,930	9,779,241	1.833	1.064 (1.044–1.085)	
Yes	Q1–Q3	322,424	4,024	3,323,349	1.211	1 (Ref.)	
	Q4	175,673	3,022	1,813,214	1.667	1.032 (0.984–1.082)	
Chronic kidney disease							0.197
No	Q1–Q3	3,279,679	34,882	33,861,284	1.030	1 (Ref.)	
	Q4	1,046,745	18,841	10,803,877	1.744	1.065 (1.045–1.085)	
Yes	Q1–Q3	89,981	1,810	928,947	1.948	1 (Ref.)	
	Q4	76,292	2,111	788,578	2.677	1.020 (0.958–1.087)	

Simplified subgroup analysis comparing Q4 vs. Q1–Q3 of the BRI for all prespecified covariates. aHR: adjusted hazard ratio; IR: incidence rate; py: person-years; Ref.: reference; MAB: medical aid beneficiary; BMI: body mass index; CKD: chronic kidney disease.

Supplementary Table S5. E-values for the principal BRI–CRC associations. The E-value represents the minimum strength of association, on the risk ratio scale, that an unmeasured confounder would need to have with both the exposure (BRI) and the outcome (CRC), above and beyond the measured covariates, to fully explain away the observed association.

Analysis	Adjusted HR (95% CI)	E-value (point)	E-value (CI limit)
Overall Q4 vs Q1	1.104 (1.075–1.135)	1.44	1.36
Male Q4 vs Q1	1.352 (1.302–1.404)	2.04	1.93
Female Q4 vs Q1*	0.891 (0.858–0.925)	1.49	1.38
Proximal colon Q4 vs Q1	1.363 (1.254–1.481)	2.07	1.82
Distal colon Q4 vs Q1	1.244 (1.166–1.328)	1.80	1.61
Rectum Q4 vs Q1	1.070 (1.011–1.132)	1.34	1.12

*For HR < 1, the E-value is calculated using 1/HR. Formula: E-value = RR + $\sqrt{[RR \times (RR - 1)]}$ for RR ≥ 1 (VanderWeele TJ, Ding P. Ann Intern Med. 2017;167:268–274). All HRs are from Model 3 (adjusted for age, sex, smoking, alcohol, exercise, income, diabetes, hypertension, hypercholesterolemia, and chronic kidney disease).

Supplementary Table S6. Ratios of hazard ratios for each interaction variable across BRI quartiles Q2, Q3, and Q4 relative to Q1, from the main quartile-based parameterization of Model 3.

Interaction variable	Q2 ratio of HR (95% CI)	Q3 ratio of HR (95% CI)	Q4 ratio of HR (95% CI)
Age 40–64 vs <40	1.154 (1.061–1.256)	1.148 (1.048–1.258)	1.161 (1.049–1.284)
Age ≥65 vs <40	1.300 (1.173–1.440)	1.267 (1.140–1.408)	1.160 (1.037–1.299)
Sex, female vs male	0.867 (0.820–0.917)	0.743 (0.704–0.785)	0.659 (0.626–0.694)
Income, low/MAB vs Q2–4	1.038 (0.969–1.111)	1.043 (0.978–1.113)	0.980 (0.920–1.043)
Smoking, former vs never	1.128 (1.038–1.225)	1.191 (1.102–1.288)	1.345 (1.246–1.451)
Smoking, current vs never	1.107 (1.038–1.181)	1.197 (1.125–1.274)	1.267 (1.192–1.348)
Alcohol, mild vs non	1.071 (1.010–1.136)	1.100 (1.040–1.163)	1.211 (1.147–1.279)
Alcohol, heavy vs non	1.021 (0.919–1.135)	1.094 (0.990–1.209)	1.162 (1.053–1.281)
Exercise, yes vs no	1.044 (0.974–1.118)	1.047 (0.981–1.118)	1.055 (0.990–1.126)
BMI ≥25 vs <25	0.986 (0.802–1.210)	1.054 (0.864–1.286)	1.228 (1.007–1.498)
Diabetes, yes vs no	1.098 (0.967–1.247)	1.094 (0.971–1.233)	1.012 (0.901–1.136)
Hypertension, yes vs no	1.039 (0.949–1.138)	1.026 (0.942–1.117)	1.047 (0.965–1.136)
Hypercholesterolemia, yes vs no	0.976 (0.884–1.077)	0.922 (0.840–1.013)	0.928 (0.848–1.015)
CKD, yes vs no	0.911 (0.789–1.052)	0.872 (0.763–0.997)	0.870 (0.768–0.985)

Model 3 adjusted for age, sex, smoking, alcohol, regular exercise, low income, diabetes, hypertension, hypercholesterolemia, and chronic kidney disease.

Supplementary Table S7. Ratios of hazard ratios for each interaction variable, from the simplified Q4-vs-Q1–Q3 parameterization of Model 3.

Interaction variable	Ratio of HR (95% CI)
Age 40–64 vs <40	1.070 (0.976–1.174)

Interaction variable	Ratio of HR (95% CI)
Age ≥65 vs <40	0.974 (0.886–1.070)
Sex, female vs male	0.788 (0.762–0.816)
Income, low/MAB vs Q2–4	0.951 (0.913–0.991)
Smoking, former vs never	1.183 (1.132–1.236)
Smoking, current vs never	1.135 (1.087–1.184)
Alcohol, mild vs non	1.136 (1.094–1.178)
Alcohol, heavy vs non	1.104 (1.038–1.174)
Exercise, yes vs no	1.018 (0.976–1.062)
BMI ≥25 vs <25	1.158 (1.111–1.206)
Diabetes, yes vs no	0.926 (0.879–0.975)
Hypertension, yes vs no	1.012 (0.972–1.054)
Hypercholesterolemia, yes vs no	0.970 (0.922–1.020)
CKD, yes vs no	0.958 (0.897–1.023)

BMI, body mass index; BRI, body roundness index; CI, confidence interval; CKD, chronic kidney disease; CRC, colorectal cancer; HR, hazard ratio; MAB, medical aid beneficiary.

Supplementary Table S8. Sensitivity analyses: extended lag periods, restricted follow-up, Fine–Gray subdistribution hazard model, and alternate BMI adjustment specifications.

Sensitivity specification	Q2 aHR (95% CI)	Q3 aHR (95% CI)	Q4 aHR (95% CI)
Primary (1-year lag)	1.030 (1.002–1.059)	1.067 (1.038–1.097)	1.104 (1.075–1.135)
2-year lag	1.030 (1.001–1.061)	1.067 (1.037–1.098)	1.108 (1.078–1.140)
3-year lag	1.035 (1.004–1.067)	1.073 (1.041–1.106)	1.116 (1.083–1.150)
5-year lag	1.046 (1.011–1.083)	1.083 (1.047–1.120)	1.129 (1.092–1.168)
Up to 5-year F/U only	1.011 (0.963–1.061)	1.051 (1.003–1.102)	1.072 (1.023–1.123)
Fine–Gray SHR	1.025 (0.996–1.054)	1.058 (1.030–1.087)	1.094 (1.065–1.124)
Model 4 (+ continuous BMI)	0.990 (0.962–1.020)	0.996 (0.965–1.027)	0.983 (0.947–1.020)
Model 5 (+ obesity, dichotomous)	1.023 (0.994–1.052)	1.043 (1.014–1.073)	1.051 (1.018–1.085)

All models adjusted for age, sex, smoking, alcohol, regular exercise, low income, diabetes, hypertension, hypercholesterolemia, and CKD. Model 4 additionally adjusts for continuous BMI; Model 5 additionally adjusts for obesity (BMI ≥25 kg/m^2) as a dichotomous covariate. Fine–Gray SHR: subdistribution hazard model with all-cause mortality and non-index-site CRC as competing events.

Supplementary Table S9a. Sex-specific BRI quartile cutoffs.

Quartile	Male (BRI range)	Female (BRI range)
Q1	≤2.63	≤2.29
Q2	2.64–3.18	2.29–3.00
Q3	3.19–3.79	3.01–3.89
Q4	≥3.80	≥3.90

Supplementary Table S9b. BRI and incident CRC risk using sex-stratified quartiles (Model

3, fully adjusted).

BRI quartile	N	Events	IR/1000 py	Model 3 aHR (95% CI)
Q1 (ref)	1,122,652	8,601	0.742	1 (Reference)
Q2	1,125,136	12,262	1.056	1.056 (1.027–1.086)
Q3	1,122,762	15,793	1.363	1.090 (1.061–1.120)
Q4	1,122,147	20,988	1.812	1.127 (1.097–1.158)

Model 3 adjusted for age, sex, smoking, alcohol, regular exercise, low income, diabetes, hypertension, hypercholesterolemia, and CKD. The Q4-vs-Q1 aHR under sex-stratified quartiles (1.127, 95% CI 1.097–1.158) is quantitatively similar to that from the pooled quartile analysis (1.104, 95% CI 1.075–1.135), confirming that the primary conclusion is robust to quartile-construction choices.
